# Healthy Eating and Physical Activity Policy, Systems, and Environmental Strategies: A Content Analysis of Community Health Improvement Plans

**DOI:** 10.3389/fpubh.2020.580175

**Published:** 2020-12-18

**Authors:** Meera Sreedhara, Melissa Goulding, Karin Valentine Goins, Christine Frisard, Stephenie C. Lemon

**Affiliations:** ^1^Division of Preventive and Behavioral Medicine, Department of Population and Quantitative Health Sciences, University of Massachusetts Medical School, Worcester, MA, United States; ^2^Clinical and Population Health Research PhD Program, Graduate School of Biomedical Sciences, University of Massachusetts Medical School, Worcester, MA, United States

**Keywords:** active transportation, physical activity, healthy eating, community health improvement plan, policy, system and environment change

## Abstract

**Background:** Policy, systems, and environmental (PSE) approaches can sustainably improve healthy eating (HE) and physical activity (PA) but are challenging to implement. Community health improvement plans (CHIPs) represent a strategic opportunity to advance PSEs but have not been adequately researched. The objective of this study was to describe types of HE and PA strategies included in CHIPs and assess strategies designed to facilitate successful PSE-change using an established framework that identifies six key activities to catalyze change.

**Methods:** A content analysis was conducted of 75 CHIP documents containing HE and/or PA PSE strategies, which represented communities that were identified from responses to a national probability sample of US local health departments (<500,000 residents). Each HE/PA PSE strategy was assessed for alignment with six key activities that facilitate PSE-change (identifying and framing the problem, engaging and educating key people, identifying PSE solutions, utilizing available evidence, assessing social and political environment, and building support and political will). Multilevel latent class analyses were conducted to identify classes of CHIPs based on HE/PA PSE strategy alignment with key activities. Analyses were conducted separately for CHIPs containing HE and PA PSE strategies.

**Results:** Two classes of CHIPs with PSE strategies emerged from the HE (*n* = 40 CHIPs) and PA (*n* = 43 CHIPs) multilevel latent class analyses. More CHIPs were grouped in Class A (HE: 75%; PA: 79%), which were characterized by PSE strategies that simply identified a PSE solution. Fewer CHIPs were grouped in Class B (HE: 25%; PA: 21%), and these mostly included PSE strategies that comprehensively addressed multiple key activities for PSE-change.

**Conclusions:** Few CHIPs containing PSE strategies addressed multiple key activities for PSE-change. Efforts to enhance collaborations with important decision-makers and community capacity to engage in a range of key activities are warranted.

## Introduction

Environmental contexts can shape diet, physical activity (PA), and subsequent chronic disease risk (1–3). Local policy, systems, and environmental (PSE) approaches can be tailored to promote sustainable opportunities for healthy eating (HE) and PA within such environments (3). PSE-change is a multisector endeavor and local health departments (LHD) are important collaborators that can support the process by leveraging knowledge of local needs, providing data analysis skills, and engaging key decision-makers (3–5). PSEs recommended for local government action to improve food environments range from zoning ordinances that permit community gardens to sugar-sweetened beverage taxes (2, 3). Evidence-based transportation systems and land use strategies that increase opportunities for PA include improving street connectivity, enhancing sidewalk and bicycle infrastructure, and developing mixed use neighborhoods (1). However, emerging research reports show that local jurisdictions variably select PSE approaches that promote cardiometabolic health (6–9).

A new paradigm of public health, Public Health 3.0, calls on local public health officials to collaborate with communities to address PSE solutions, but moving an issue onto the agenda is challenging due to barriers ranging from lack of political and community support to limited staff capacity and knowledge (4, 10–13). An established obesity prevention framework has identified six key activities public health practitioners and communities can undertake to overcome barriers and facilitate PSE-change (3). The key activities include identifying and framing the problem, engaging and educating key people, identifying PSE solutions, utilizing available evidence, assessing social and political environment, and building support and political will. Although this framework offers a guide to navigate this complex process, the extent to which PSE strategies are developed in alignment with these key activities is unknown.

Strategic health planning approaches such as community health improvement plans (CHIPs) can help advance PSE-change initiatives. CHIPs aim to develop objectives and select strategies in response to local needs through collaborative, systematic and data-driven approaches (14). These approaches have also been identified as necessary to support HE and PA PSE strategies (2, 3). Communities and LHDs are increasingly engaging in population health activities, including health improvement planning, potentially due to benefits such as reductions in diabetes and cardiovascular disease mortality (15, 16). Although most CHIPs generally address nutrition and PA, emerging evidence suggests that strategic health improvement plans underutilize PSE strategies to address HE and PA (17–20).

The Public Health Accreditation Board and the National Association of County and City Health Officials recommend that public health officials select evidence- and policy-based strategies for inclusion in a CHIP whereas the Centers for Disease Control and Prevention's Sustainability Planning Guide addresses multiple key activities for PSE-change (21–23). To our knowledge, no study has been conducted to assess HE and PA PSE strategies included in CHIP documents. Therefore, the objective of this study was to describe types of HE and PA strategies included in CHIPs and assess strategies designed to facilitate successful PSE-change using an established framework in which six key activities to catalyze change have been identified.

## Methods

### Study Design

This cross-sectional content analysis of CHIP documents was approved by the University of Massachusetts Medical School Institutional Review Board (24).

### Sample

A convenience sample of CHIPs was selected from communities represented by respondents to a 2017 probability survey of United States (US) LHDs serving fewer than 500,000 residents (response rate 30%, *n* = 209) (20). Among respondents, 93 (44%) LHDs reported participating in a CHIP within the past 5 years that included one or more of 13 HE policy strategies or eight strategies supportive of active transportation. CHIP document searches occurred via internet, e-mail, and telephone between July 2018–February 2019. We excluded 18 communities for which CHIPs could not be located (*n* = 7); lacked any food or PA-related strategy (*n* = 8); and among three pairs of communities that shared a CHIP, we randomly selected one community for CHIP document attribution and excluded the other. Seventy-five CHIP documents met eligibility criteria of being developed between 2012–2017 and containing a strategy related to food and/or PA.

### LHD Characteristics

Characteristics of LHDs were collected during the previous survey (20). Characteristics included: US census geographic region (South, West, Northeast, Midwest), population size served (<25,000, 25,000–49,999, 50,000–99,999, 100,000–499,999 residents), structure (municipal, county or city-county, other), state and LHD governance (decentralized or centralized/shared/mixed) and Public Health Accreditation Board (PHAB) status (not accredited, achieved accreditation, or accreditation in progress or planned).

### Data Collection Tool Development

Literature and resources guided the development of a standardized data collection tool and codebook (3, 17, 21–23, 25, 26). The tool was iteratively revised based on study team and expert feedback; cognitive interviews with LHD officials and experts (*n* = 4); and pilot testing (*n* = 14 CHIPs). The tool was organized into four domains related to general CHIP characteristics, priority areas, objectives, and HE and PA strategies.

#### General CHIP Characteristics

The first domain collected general CHIP characteristics important for strategic health planning. We created dichotomous variables to indicate whether each of the following characteristics was stated in the CHIP document and additionally created categorical variables from text descriptions describing characteristics (Table 1): strategic planning framework; systematic assessment of data; alignment with external priorities; and evaluation and dissemination. Additionally, we recorded if at least one category of collaborator or participant was described as assisting with CHIP development or implementation (17).

**Table 1:** Characteristics of CHIP documents among all study CHIPs (*n* = 75) and CHIPs with at least one policy, systems, or environmental healthy eating or physical activity strategy (*n* = 51).

**CHIP Characteristics**	**% (*****n*****)**	
	**All study CHIPs (*n* = 75)**	**CHIPs with >1 PSE strategy (*n* = 51)**
Strategic program planning framework applied	89.3% (67)	92.2% (47)
Mobilizing for Action through Planning and Partnerships	48.0% (36)	49.0% (25)
Elements of an unspecified framework	24.0% (18)	25.5% (13)
County Health Rankings Model	8.0% (6)	7.8% (4)
Association for Community Health Improvement/ Health Research & Educational Trust framework	6.7% (5)	7.8% (4)
Other	18.7% (14)	19.6% (10)
Systematic assessment informed CHIP development	96.0% (72)	96.1% (49)
Quantitative health indicator data collected	84.0% (63)	84.3% (43)
Community resident input on health priorities	52.0% (39)	54.9% (28)
Other MAPP assessments: community themes and strengths, local public health system, & forces of change assessments	29.3% (22)	27.5% (14)
Organizational capacity assessment	4.0% (3)	3.9% (2)
Strengths, weaknesses, opportunities, and threats assessment	8.0% (6)	9.8% (5)
No description of data collected for systematic assessment	8.0% (6)	9.8% (5)
Alignment with external priorities	82.7% (62)	86.3% (44)
Local	20% (15)	21.6% (11)
State	61.3% (46)	62.8% (32)
Regional	4.0% (3)	5.9% (3)
National	56.0% (42)	58.8% (30)
Other	4.0% (3)	3.9% (2)
Evaluation and Dissemination	85.3% (64)	84.3% (43)
Evaluation or monitoring plan described for overall CHIP or individual priority area, objective, and/or strategy	62.7% (47)	64.7% (33)
Time points for evaluation or monitoring specified	53.3% (40)	49.0% (25)
Dissemination plan for evaluation findings specified (e.g., report out meetings, posting updated data or evaluation on a website, publishing a progress report)	40.0% (30)	36.9% (20)
Funding or staff resources identified	4.0% (3)	5.9% (3)
Unspecified (e.g., general statement that progress will be evaluated regularly)	16.0% (12)	19.6% (10)
Select list of collaborator or participant categories		
Hospital or health care organization	97.3% (73)	98.0% (50)
Hunger relief organization	36.0% (27)	35.3% (18)
Food related group	60.0% (45)	58.8% (30)
Agriculture	26.7% (20)	31.4% (16)
Physical Activity related group	37.3% (28)	45.1% (23)
Department of transportation, public works, or engineering	40.0% (30)	49.0% (25)^*^
Department of land use planning	29.3% (22)	35.3% (18)
Department of community or economic development	13.3% (10)	11.8% (6)
Department of parks and recreation	37.3% (28)	35.3% (18)
City manager, town manager, county manager or county administrator	14.7% (11)	15.7% (8)

LHD, local health department; CHIP, community health improvement plan. Percentages may not equal 100% due to rounding; Categories are not mutually exclusive; Chi-square and Fisher's exact tests compared CHIPs containing at least one HE or PA PSE strategy and CHIPs without a PSE strategy;

* *Fisher's exact test p = 0.02*.

#### Priority Areas

The second domain focused on priority areas, defined as key topics determined in a community planning process (21), that contained strategies supportive of HE and/or PA. We derived the following categories from stated descriptions to describe how HE and PA were prioritized in CHIPs: obesity; chronic disease; food and/or PA; health equity, social determinants of health other than access to healthy food or the built environment (e.g., education, access to care), and specific populations (e.g., maternal health). Categories were not mutually exclusive.

#### Objectives

The third domain collected the total number and stated descriptions of objectives, defined as statements that describe the outcome to be achieved, under which HE and/or PA strategies were listed (25). For each objective, we assessed if individual SMART criteria were met (specific, measurable, achievable, realistic/relevant, or time-phased) (25). Supplementary Table 1 lists definitions and examples.

#### HE and PA Strategies

The final domain captured the total number and stated descriptions of HE and PA strategies, defined as a “collection of actions which has a reasoned chance of achieving desired objectives” (23). Duplicate strategies were evaluated once. We excluded strategies with equivocal evidence for HE, PA, or obesity-prevention related to breastfeeding, paratransit, fall prevention programs, and strategies where it was unclear if food or PA was the primary focus (e.g., school wellness policies).

To evaluate HE/PA strategies, we determined whether it was a policy, system, environmental and/or non-PSE approach (e.g., educational program). We generated a dichotomous variable to indicate whether the strategy was a PSE. We assessed whether each PSE strategy was aligned with individual key activities for PSE-change that this study used as a framework (3). This resulted in six dichotomous variables for each PSE strategy. Supplementary Table 1 lists definitions and examples.

### Interrater Reliability

To enhance reliability, two coders (MS & MG) used the tool and codebook to independently code a random sample of CHIP documents (*n* = 15, 20%). The average interrater reliability (IRR) (92.0%) and Kappa (0.8399) were strong (27). Additionally, coders independently assessed all PSE strategies (*n* = 186) for alignment with the six key activities (IRR = 97.3%; Kappa = 0.9356). Disagreements were discussed until consensus was met or resolved by study team members (SCL & KVG).

### Statistical Analysis

Descriptive statistics of CHIP, priority area, and objective and strategy characteristics were calculated. Chi-square and Fisher's exact tests were used to assess selection bias by comparing (a) LHD characteristics for analyzed CHIPs (*n* = 75) to those excluded (*n* = 18) and (b) CHIP characteristics for CHIPs with any HE/PA PSE strategy (*n* = 51) to those without (*n* = 24). Stata/MP 13.1 statistical software was used.

To describe the development of objectives and HE/PA-related PSE strategies across all CHIPs, we generated standardized sub-scores for SMART criteria (objective sub-score) and key activities for PSE-change (strategy sub-score) by topic area. The total number of key activities that each HE/PA-related PSE strategy met was summed and averaged for each CHIP. We repeated this for the objective sub-score. To standardize sub-scores, we applied the same range (0–1) using the min-max approach of subtracting the minimum value from the average sub-score and dividing by the range (28). Sub-scores were set to zero for CHIPs that did not contain any objectives or HE/PA-related PSE strategies.

Traditional and multilevel latent class analyses together provide a comprehensive understanding of both PSE strategies and CHIPs (29). Analyses were conducted separately by topic area. Traditional latent class analyses identified classes of PSE strategies, which described patterns of alignment with key activities. Multilevel latent class analyses accounted for clustering of PSE strategies within CHIPs and identified distinct classes of CHIPs, which illustrated distributions of strategy patterns within classes of CHIPs. A non-parametric approach was used because the key activities were dichotomous indicators. Models included random effects which allowed for random intercepts and slopes to vary across clusters of CHIPs.

Models with varying numbers of classes (one to five classes) were estimated. During traditional latent class analyses, fit statistics (e.g., Bayesian Information Criteria), interpretability of classes, and likelihood ratio tests guided model selection. During multilevel latent class analyses, model fit statistics and interpretability of classes guided model selection. We calculated item-response probabilities, which indicated the likelihood that PSE strategies aligned with specific key activities. Mplus (Version 8) statistical software was used.

## Results

We analyzed 75 CHIP documents. Characteristics of LHDs associated with CHIPs analyzed in our study (*n* = 75) and those excluded (*n* = 18) were similar with respect to geographic region, population size served, structure, or state governance. However, more excluded CHIPs were developed with assistance from a LHD that was not accredited by PHAB (67%) than CHIPs that were analyzed (31%) (*p* = 0.03). We found no differences between CHIPs with or without HE/PA PSE strategy (*n* = 51 and *n* = 24, respectively) (Table 2).

**Table 2:** Characteristics of LHDs participating in the development of a CHIP among all study CHIPs (*n* = 75) and CHIPs with at least one policy, systems, or environmental healthy eating or physical activity strategy (*n* = 51).

**LHD Characteristics**	**% (*****n*****)**	
	**All study CHIPs (*n* = 75)**	**CHIPs with PSEs (*n* = 51)**
US Census Geographic Region		
South	21.3% (16)	17.7% (9)
West	26.7% (20)	29.4% (15)
Northeast	24.0% (18)	23.5% (12)
Midwest	28.0% (21)	29.4% (15)
Population Size of LHD Service Area^*^		
<25,000	20.3% (15)	24.0% (12)
25,000–49,999	21.6% (16)	22.0% (11)
50,000–99,999	24.3% (18)	20.0% (10)
100,000–499,999	33.8% (25)	34.0% (17)
Structure^*^		
Municipal (city or town)	12.2% (9)	10.0% (5)
County or City-county	77.0% (57)	76.0% (38)
Other (State-run, regional, other)	10.8% (8)	14.0% (7)
State and LHD Governance		
Centralized, shared, mixed	21.3% (16)	21.6% (11)
Decentralized	78.7% (59)	78.4% (40)
Public Health Accreditation Board status^†^		
Not accredited	30.7% (23)	37.3% (19)
Achieved accreditation	24.0% (18)	27.5% (14)
Accreditation in progress or planned	45.3% (34)	35.3% (18)

LHD, local health department; CHIP, community health improvement plan; PSE, policy, systems, and environmental strategy. Percentages may not equal 100% due to rounding; Fisher's exact tests compared CHIPs containing at least one HE or PA PSE strategy and CHIPs without a PSE strategy;

* *One LHD did not provide a response to population size served or structure; Fisher's exact test p = 0.04*.

### General Characteristics

#### LHD Characteristics

Table 2 contains a description of LHD characteristics for all study CHIP documents and those containing any HE or PA PSE strategy. Among all study CHIPs, most were developed with assistance from a LHD that was county or city-county based (77%), decentralized (i.e., locally governed) (79%), represented 50,000 to 499,999 residents (58%), and had either achieved national accreditation from PHAB (24%) or were in pursuit of accreditation (45%).

#### CHIP Document Characteristics

Table 1 contains a description of CHIP characteristics for all study CHIPs as well as those containing at least one HE/PA PSE strategy. Among all study CHIPs, most indicated the CHIP was developed using a strategic planning framework (89%), with Mobilizing for Action through Planning and Partnerships being the most common (48%). Many used systematic assessment data (96%) and stated alignment with state or national priorities (61 and 56%, respectively). Most CHIPs mentioned evaluation or dissemination (85%), but 40% stated a specific plan to disseminate findings and 4% identified resources to carry out such activities. Hospitals or health care organizations were the most often reported CHIP collaborator category (97%). Conversely, advocacy or service groups related to food (60%) and PA (37%) and government departments such as land use planning (29%) were less frequently reported.

### Priority Areas

Among the 75 study CHIP documents, four did not contain any priority areas or the relationship between strategies and priority areas was unspecified and precluded categorization. HE strategies in the CHIPS were prioritized under categories of HE (26% of CHIPs), obesity (23%), and chronic disease (27%). Similar proportions were observed for CHIPs with PA strategies (PA: 21%, obesity: 24%, chronic disease: 29%). Few CHIPs prioritized strategies under more nuanced categories such as built environment, injury prevention, and other social determinants of health/health equity.

### Objectives

Nine CHIPs did not contain any objectives related to HE or PA strategies. Most of the 66 CHIPs containing objectives related to a HE or PA strategy were clearly formatted so the reader was able to determine the link between a specific strategy and the objective it was meant to achieve. This was not the case for 25 CHIPs. Table 3 reports descriptive statistics by topic area. Objective sub-scores were similarly high for each topic area (HE: mean 0.62, SD 0.39, median 0.77; PA: mean 0.61, SD 0.39, median 0.74).

**Table 3:** Characteristics of objectives among CHIPs with objectives related to healthy eating or physical activity, mean (SD).

**Characteristics**	**Healthy eating**	**Physical activity**
	**(*n* = 65 CHIPs)**	**(*n* = 61 CHIPs)**
Total number of objectives	3.5 (3.3), Range 1–21	3.8 (3.8), Range 1–21
All SMART criteria met	2.0 (3.0)	2.2 (3.0)
Specific	3.0 (3.4)	3.3 (3.9)
Measurable	2.5 (3.2)	2.8 (3.5)
Achievable	2.1 (3.1)	2.4 (3.3)
Realistic/relevant	2.2 (3.1)	2.4 (3.4)
Time-phased	2.6 (3.3)	2.9 (3.3)

*CHIP, community health improvement plan; SD, standard deviation*.

### Strategies

CHIPs averaged six HE (SD 6.4) and six PA (SD 7.2) strategies. A greater mean number of strategies focused on the individual or interpersonal-level (HE: 5.0, SD 6.1; PA: 4.5, SD 6.0) than PSE changes. Study CHIPs averaged one HE PSE strategy (SD 1.2) and two PA PSE strategies (SD 2.8). More than half of CHIPs included at least one PSE strategy (HE: 55%; PA: 61%).

Alignment of PSE strategies with key activities that facilitate PSE-change varied by activity. Nearly all CHIPs with a PSE strategy aligned with the activity termed identifying PSE solutions (HE: 98%; PA: 100%) and contained, on average, 1.9 (SD 1.1) HE and 3.0 (SD 3.0) PA strategies that addressed this activity. The next most common activity that CHIPs addressed was building support and political will (HE: 43%, mean 0.6, SD 0.7; PA: 58%, mean 0.9, SD 0.9). One-third of CHIPs addressed identifying and framing the problem (HE: 35%, mean 0.4, SD 0.6; PA: 37%, mean 0.7, SD 1.1). Neither utilizing available evidence (HE: 25%, mean 0.4, SD 0.9; PA: 35%, mean 0.9, SD 1.1) nor engaging and educating key people (HE: 18%, mean 0.2, SD 0.5; PA: 23%, mean 0.3, SD 0.7) were commonly addressed activities. Assessing social and political environment was the least commonly reported strategy (HE: 3%, mean 0.0, SD 0.2; PA: 2%, mean 0.1, SD 0.5). Strategy sub-scores were similarly low by topic area (HE: mean 0.13, SD 0.24, median 0; PA: mean 0.17, SD 0.24, median 0).

### Latent Class Analyses

A total of 76 HE PSE strategies from 40 CHIPs and 129 PA PSE strategies from 43 CHIPs were assessed in latent class analyses.

#### Classes of Strategies

**Figure 1:**
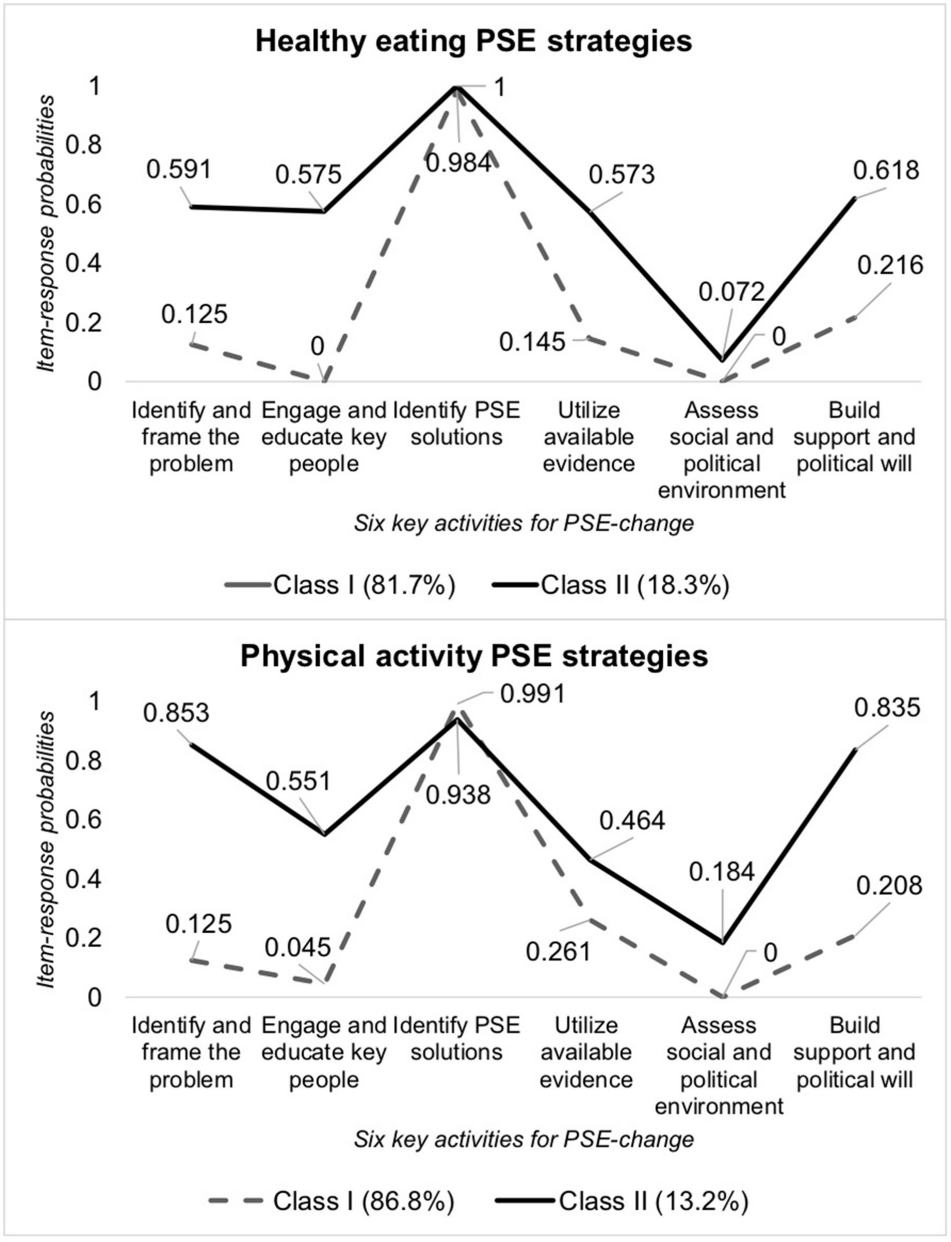
Item-response probabilities of PSE strategy alignment with key activities by strategy class. PSE, policy, systems, and environmental approaches.

Traditional latent class analyses for each topic area identified two distinct classes of PSE strategies. The selected models exhibited the lowest Bayesian information criterion and good entropy. Tests of two vs. three classes were not significant (Supplementary Table 2). Figure 1 depicts item-response probabilities for each class of PSE strategies by topic area. Most PSE strategies were grouped into Class I (HE: 81.7%; PA: 86.8%), which exhibited high item-response probabilities for the key activity identifying PSE solutions. An example of a PA PSE strategy grouped in Class I is:

“*Adopt policies such as Complete Streets.”*

A small proportion of PSE strategies were grouped into Class II (HE: 18.3%; PA: 13.2%), which comprehensively addressed multiple key activities. An example of a PA PSE strategy that was grouped in Class II is:

“*Develop Complete Street policy, implementation. Action steps: Identify project under discussion.; Identify locations.; Contact planning, zoning, depts. in municipalities to determine status of policy implementation. Look at Canada/Europe as models.; Conduct Street Audits.; Look for community interest for street audits (i.e., [community organizations and local health officers]).; Explain ‘Complete Streets’ to community groups in order to obtain input, locations; Hold focus groups at senior centers, faith-based organizations to determine needs.”*

Item-response probabilities for Class II indicate that these PSE strategies addressed a wider range of key activities than PSE strategies found in Class I. PSE strategies in Class II exhibited high item-response probabilities for five out of six key activities. PA PSE strategies in Class II had higher probabilities for the activities of identifying and framing the problem and building support and political will than HE PSEs in the same class. PSE strategies across all classes and topic areas exhibited low probabilities for assessing social and political environment.

#### Classes of CHIPs

Multilevel latent class analyses, which accounted for strategy clustering within CHIPs, identified two classes of CHIPs for both HE and PA. Most CHIPs (HE: 75%; PA: 79%) were grouped together in Class A, which were mainly composed of Class I PSE strategies that simply identified a PSE solution (HE: 99.9% of PSE strategies in Class A; PA: 100% of PSE strategies in Class A). A smaller proportion of CHIPs were categorized into a separate group termed Class B (HE: 25%; PA: 21%). Class B CHIPs were mostly composed of Class II PSE strategies that addressed a wide range of key activities (HE: 79.7%; PA: 90.3%), and the remainder of PSE strategies contained in Class B CHIPs simply identified a PSE solution (HE: 20.3%; PA 9.7%).

LHD and CHIP characteristics were similar for each CHIP class and by topic area (Table 4). The only significant difference observed was among CHIPs with PA PSEs where more Class B CHIPs (66.7%) stated collaboration with a department of land use planning when compared to Class A CHIPs (29.4%) (*p* = 0.06).

**Table 4:** Characteristics of study CHIP classes by topic area.

	**Healthy eating (*****n*** **=** **40), % (*****n*****)**		**Physical activity (*****n*** **=** **43), % (*****n*****)**	
	**Class A (*n* = 30)**	**Class B (*n* = 10)**	**Class A (*n* = 34)**	**Class B (*n* = 9)**
**LHD CHARACTERISTICS**				
US Census geographic region				
South	13.3% (4)	10.0% (1)	17.7% (6)	11.1% (1)
West	33.3% (10)	20.0% (2)	35.3% (12)	22.2% (2)
Northeast	23.3% (7)	30.0% (3)	17.7% (6)	44.4% (4)
Midwest	30.0% (9)	40.0% (4)	29.4% (10)	22.2% (2)
Population size served^*^				
<25,000	20.7% (6)	20.0% (2)	17.7% (6)	25.0% (2)
25,000–49,999	20.7% (6)	20.0% (2)	23.5% (8)	25.0% (2)
50,000–99,999	20.7% (6)	30.0% (3)	20.6% (7)	25.0% (2)
100,000–499,999	37.9% (11)	30.0% (3)	38.2% (13)	25.0% (2)
Structure^*^				
Municipal (city or town)	10.3% (3)	20.0% (2)	11.8% (4)	12.5% (1)
County or City-county	79.3% (23)	70.0% (7)	70.6% (24)	75.0% (6)
Other (State-run, regional, other)	10.3% (3)	10.0% (1)	17.7% (6)	12.5% (1)
State and LHD Governance				
Centralized, shared, or mixed	16.7% (5)	20.0% (2)	23.5% (8)	11.1% (1)
Decentralized	83.3% (25)	80.0% (8)	76.5% (26)	88.9% (8)
Public Health Accreditation Board status				
Not accredited	26.7% (8)	60.0% (6)	29.4% (10)	55.6% (5)
Achieved accreditation	33.3% (10)	10.0% (1)	38.2% (13)	11.1% (1)
Accreditation in progress or planned	40.0% (12)	30.0% (3)	32.4% (11)	33.3% (3)
**CHIP CHARACTERISTICS**				
Strategic program planning framework applied	96.7% (29)	90.0% (9)	91.2% (31)	88.9% (8)
Mobilizing for Action through Planning and Partnerships	60.0% (18)	20.0% (2)	47.1% (16)	33.3% (3)
Elements of an unspecified framework	26.7% (8)	30.0% (3)	26.5% (9)	22.2% (2)
County Health Rankings Model	6.7% (2)	10.0% (1)	8.8% (3)	11.1% (1)
Association for Community Health Improvement/ Health Research & Educational Trust framework	6.7% (2)	20.0% (2)	5.9% (2)	22.2% (2)
Other	20.0% (6)	10.0% (1)	26.5% (9)	11.1% (1)
Systematic assessment informed CHIP development	96.7% (29)	100.0% (10)	94.1% (32)	100.0% (9)
Quantitative health indicator data collected	83.3% (25)	90.0% (9)	79.4% (27)	88.9% (8)
Community resident input on health priorities	56.7% (17)	60.0% (6)	58.8% (20)	44.4% (4)
Other MAPP assessments: community themes and strengths, local public health system, & forces of	30.0% (9)	20.0% (2)	23.5% (8)	22.2% (2)
change assessments				
Organizational capacity assessment	3.3% (1)	0.0% (0)	0.0% (0)	11.1% (1)
Strengths, weaknesses, opportunities, and threats assessment	6.7% (2)	10.0% (1)	11.8% (4)	11.1% (1)
No description of data collected for systematic assessment	10.0% (3)	10.0% (1)	11.8% (4)	11.1% (1)
Alignment with external priorities	93.3% (28)	70.0% (7)	85.3% (29)	77.8% (7)
Local	23.3% (7)	0.0% (0)	26.5% (9)	11.1% (1)
State	73.3% (22)	60.0% (6)	61.8% (21)	66.7% (6)
Regional	6.7% (2)	0.0% (0)	2.9% (1)	0.0% (0)
National	66.7% (20)	50.0% (5)	58.8% (20)	55.6% (5)
Other	6.7% (2)	0.0% (0)	5.9% (2)	0.0% (0)
Evaluation and Dissemination	93.3% (28)	80.0% (8)	79.4% (27)	88.9% (8)
Evaluation or monitoring plan described for overall CHIP or individual priority area, objective, and/or	63.3% (19)	70.0% (7)	61.8% (21)	77.8% (7)
strategy				
Time points for evaluation or monitoring specified	56.7% (17)	50.0% (5)	41.2% (14)	55.6% (5)
Dissemination plan for evaluation findings specified (e.g., report out meetings, posting updated data	40.0% (12)	30.0% (3)	44.1% (15)	33.3% (3)
or evaluation on a website, publishing a progress report)				
Funding or staff resources identified	6.7% (2)	10.0% (1)	0.0% (0)	11.1% (1)
Unspecified (e.g., general statement that progress will be evaluated regularly)	30.0% (9)	10.0% (1)	23.5% (8)	0.0% (0)
Select list of collaborator or participant categories				
Hospital or health care organization	100.0% (30)	100.0% (10)	97.1% (33)	100.0% (9)
Hunger relief organization	43.3% (13)	30.0% (3)	44.1% (15)	22.2% (2)
Food related group	56.7% (17)	80.0% (8)	52.9% (18)	66.7% (6)
Agriculture	33.3% (10)	50.0% (5)	32.4% (11)	33.3% (3)
Physical Activity related group	33.3% (10)	70.0% (7)	47.1% (16)	66.7% (6)
Department of transportation, public works, or engineering	53.3% (16)	60.0% (6)	50.0% (17)	66.7% (6)
Department of land use planning	33.3% (10)	60.0% (6)	29.4% (10)^†^	66.7% (6)^†^
Department of community or economic development	13.3% (4)	20.0% (2)	11.8% (4)	22.2% (2)
Department of parks and recreation	36.7% (11)	50.0% (5)	38.2% (13)	33.3% (3)
City manager, town manager, county manager or county administrator	20.0% (6)	10.0% (1)	20.6% (7)	0.0% (0)

CHIP, community health improvement plan; LHD, local health department; MAPP, Mobilizing for Action through Planning and Partnerships. Percentages may not equal 100% due to rounding; CHIP characteristic categories are not mutually exclusive; Chi-square and Fisher's exact test compared Classes of CHIPs;

* One LHD did not provide a response to population size served or structure;

† *Fisher's exact test p = 0.06*.

## Discussion

We conducted a content analysis of CHIP documents that contained HE and PA strategies to identify patterns of strategy and CHIP alignment with key activities that facilitate successful PSE change. We identified two classes of CHIP documents. Class A CHIPs mostly contained PSE strategies that simply identified a PSE solution. Class B consisted of a smaller proportion of CHIPs that were characterized by PSE strategies that comprehensively addressed multiple key activities.

To our knowledge, this is the first study that has been conducted to assess PSE strategies related to HE or PA included in CHIPs. We offer two explanations for our findings. First, national guidance for developing PSE strategies that address multiple key activities that catalyze PSE-change is limited. National public health authorities such as the Public Health Accreditation Board recommend that LHDs identify PSE solutions and use available evidence when selecting strategies for inclusion in a CHIP (21). However, few resources are available to guide communities and local public health systems through the development of comprehensive PSE strategies for inclusion in a CHIP that address additional key activities (21–23). Community workshops, trainings, and technical assistance have successfully guided communities through the process of multiple key activities to produce PSE-change (30, 31). Alternatively, details about the strategies in our sample of CHIPs may be outlined elsewhere. For example, interagency coordination and community engagement may be detailed in a formal Complete Streets policy, separate from the CHIP.

PSE strategies in our study were least aligned with the activity of assessing social and political environment. Assessing and mobilizing public and political support is critical for successful PSE-change but uncommon (32, 33). A 2014 survey of opinion leaders and the general public in Kansas found that perceptions and beliefs about obesity predicted support for policies related to HE and PA (34). A case study of a successful sugar-sweetened beverage ballot measure in San Francisco cited qualitative assessment of public interest in regulations as directly informing policy deliberations that were previously unsuccessful (35). Conducting and supporting this activity requires a multidisciplinary coalition of actors (33). Taking steps toward assessing social and political environments may also address local context (e.g., economics, political climate and support), which can influence PSE implementation (31).

CHIPs contained more PA-related PSE strategies, which exhibited greater alignment with key activities to facilitate change than HE PSE strategies. These differences could not be formally tested because CHIPs often contained strategies from both topic areas, but may be explained because stronger evidence supports PA PSE approaches (1). The unique and contextually specific barriers (e.g., lobbying) that accompany HE PSEs may hinder selection and explain our findings (12, 32).

Changing food and activity environments requires strong diverse engagement from a multidisciplinary coalition of actors, which was not observed in our study. Previous public health and strategic health planning studies report similar lack of collaboration (16–18). Communities in our study may not have perceived the need to engage collaborators such as land use planners, potentially because mostly individual or interpersonal strategies were selected. However, such collaborations were also not common in CHIPs with a PSE strategy that would benefit from such partnership. Communities that support health improvement planning activities through dense collaborative networks experience increased PSE-change and reduced chronic disease mortality (2, 3, 15, 31). Yet cross-sector collaborations are challenging and thus require further investigation.

CHIPs in our sample largely addressed food or PA through individual-level strategies such as education and clinical interventions. An analysis of PA content in US state-level obesity-related plans also found greater attention paid toward individual and interpersonal strategies instead of changes to the built environment (18). Our observation mirrors global trends where intervening on behavior continues to be the focus, despite widespread knowledge that multilevel interventions are necessary to address obesity (36).

## Limitations and Strengths

Our findings should be considered alongside the study's limitations and strengths. We identified our sample from national probability survey data, but this was ultimately a convenience sample, and the findings may not be representative of all US CHIPs serving fewer than 500,000 residents. Our estimates may be inaccurate because CHIP documents could lack or overstate important details. For example, a strategic planning framework may have informed CHIP development but was not stated in the document. Nevertheless, many of the documents were public records and were developed with assistance from nationally accredited LHDs or those pursuing accreditation, which may have compelled communities to accurately report the CHIP development process. Creating a reliable data collection tool was difficult because the CHIP documents varied widely with respect to content, format, and terminology used. However, we believe we enhanced reliability through interrater reliability processes and by developing a standardized codebook and tool based on literature, expert feedback, and pilot testing. A sensitivity analysis to assess for selection bias found no difference in characteristics of LHDs participating in CHIP development for CHIPs that were analyzed or excluded with the exception of PHAB accreditation status. Despite these limitations, we believe this study is novel because it is the first to assess food and PA strategies included in CHIPs. We also used innovative methods to investigate patterns of PSE strategy alignment with key activities supportive of PSE-change, filling an important gap in the literature.

## Conclusion

Through the current study, we identified opportunities for greater alignment of PSE strategies with key activities that support PSE-change. Additionally, interpretation of the findings suggests there remains room for improvement in collaborations with important decision-makers during the CHIP development process.

## Data Availability Statement

The raw data supporting the conclusions of this article will be made available by the authors, without undue reservation.

## Ethics Statement

The studies involving human participants were reviewed and approved by University of Massachusetts Medical School Institutional Review Board. Written informed consent for participation was not required for this study in accordance with the national legislation and the institutional requirements.

## Author Contributions

MS conceptualized the study, developed the data collection tool, conducted data collection, coding and analysis, interpreted the data, and drafted the manuscript. MG conducted coding and revised the manuscript critically for important intellectual content. KVG contributed to study conceptualization, provided input on coding, and revised the manuscript critically for important intellectual content. CF provided input on the analysis and interpretation and revised the manuscript critically for important intellectual content. SL provided input on study conceptualization, data collection tool development, data collection, coding, analysis, interpretation, and revised the manuscript critically for important intellectual content. All authors contributed to the article and approved the submitted version.

## Conflict of Interest

The authors declare that the research was conducted in the absence of any commercial or financial relationships that could be construed as a potential conflict of interest.
