# Supplementary material for: Healthy Eating and Physical Activity Policy, Systems, and Environmental Strategies: A Content Analysis of Community Health Improvement Plans
Source: Front Public Health. 2020 Dec 18;8:580175. doi: 10.3389/fpubh.2020.580175 (PMC7775553; doi:10.3389/fpubh.2020.580175)
Supplement: Supplementary file 1 [file Table_1.docx]

**Supplemental Digital Content Table 1. Definitions of objective and strategy characteristics from literature and examples from CHIP documents with healthy eating and/or physical activity strategies**

| **Characteristics** | **Definitions & Examples** |
| --- | --- |
|  |  |
| **OBJECTIVES^25^** | |
| **Specific** | Specifies what action or activity will be accomplished and for whom.   - *By 2020, reduce the adolescent obesity rate in [County] from 20% to 18%. (HE)* |
| **Measurable** | Written using quantifiable terms and often indicates how success will be measured or how much change will be expected.   - *By 2020, increase the percent of [County] youth who engage in the recommended amount of physical activity of 60 minutes a day, 7 days a week, from 27% to 35%. (PA)* |
| **Achievable** | Realistically accomplished given current resources and constraints. Compare with an established target set by Healthy People 2020 or other credible resources if possible.   - *Reduce food insecurity among [County] households with children from 21.7 percent to 19.5 percent (HE)* |
| **Realistic/ Relevant** | Contributes to the overarching goal or strategy or addresses the scope of the health program and proposes reasonable programmatic steps.   - *By December 2017, the [School District] will adopt a Student Wellness Policy that meets state and federal requirements for nutrition and physical activity. (HE & PA)* |
| **Time-phased** | Provides a timeline indicating when the objective will be met or achieved.   - *By December 31, 2018, increase by 15% the number of adults with access to schools for physical activity with joint use agreements that are inclusive to and publicized throughout their community. (PA)* |
| **STRATEGIES** | |
| **Non-PSE** | Program or intervention that is not a PSE change-initiative.   - *Provide nutrition related education to patients. (HE)* - *Develop free community fitness programs and community-wide physical activity campaigns. (PA)* |
| **Any PSE strategy** | Any strategy intending to create a policy, systems, and/or an environmental change |
| **Policy^26^** | Legislative or organizational laws, regulations, rules, protocols, and procedures, ordinance or bylaw, plan, design standards, reallocation of existing/new funding, or tax to be implemented by local jurisdiction.   - *Support full implementation of a community garden master plan. (HE)* - *Encourage local government to adopt at least one new ordinance that mandates new streets shall be designed to enhance traffic safety for bicyclists and pedestrians. (PA)* |
| **System^26^** | Change that impacts all aspects of an organization, institution, or system.   - *Implement nutrition and beverage standards in public institutions, worksites, and other key locations such as hospitals. (HE)* - *Build and sustain a Safe Routes to School programs with each school district. (PA)* |
| **Environment^26^** | Change made to the physical built environment.   - *Start a community garden in a low-income community. (HE)* - *Create/promote neighborhood greenways. (PA)* |
| **Six key activities that facilitate PSE change^3^** | |
| **Identify and frame the problem** | Presenting the issue as a problem worthy of action and caused by factors amenable to policy intervention.  Adaptation made to the Lyn et al. framework definition: Strategy is assessed as aligned if it states the specific problem or issue to be addressed.   - *Use best practice worksite wellness policies that increase availability of healthy beverages and limit employer provision of sugary beverages at worksites. (HE)* - *Establish bus routes or other transit options in unserved communities as needed based on transportation gap study. (PA)* |
| **Engage and educate key people** | Intentional interaction with politicians, public officials, and stakeholders to raise awareness of the problem and initiate public and political engagement.   - *Establish [a] Food Policy Council/Network to promote coordination among partners and advance policy initiatives related to healthy food access (HE)* - *Establish a district-wide Safe Routes to School task force for ongoing identification and implementation of systems, policies, and school-level changes to support increased walking and biking to school. (PA)* |
| **Identify PSE solutions** | Defining policy opportunities and specific levers to influence food systems or physical activity environments.  Adaptation made to the Lyn et al. framework definition: Strategy is also assessed as aligned if it relates to advocating, supporting, identifying, adopting, and/or implementing a PSE-change initiative.   - *Start a community garden in a low-income community. (HE)* - *Adopt a Complete Streets Policy (PA)* |
| **Utilize available evidence** | Using credible sources of evidence to inform policy formulation.  Adaptation made to the Lyn et al. framework definition: Strategy is also assessed as aligned if it cites a source of evidence related to the effectiveness of the strategy.   - *Adopt revised health-conscious policies for competitive foods (those sold in vending machines), food sold at sports events, bake sales, celebrations, etc. [CHIP cited http://www.countyhealthrankings.org/policies/school-nutrition-standards] (HE)* - *Seek to advance our communities by incorporating “Active Design” in our parks, trails, and other recreational facilities. [CHIP cited https://centerforactivedesign.org] (PA)* |
| **Assess social and political environment** | Working to understand the prevailing context in which policy proposals are considered for adoption.   - *Increase the number of municipalities and community-based organizations in [the] County that have adopted food procurement standards and policies based on the Dietary Guidelines for Americans by 10…Activities: Develop and conduct a survey of community-based organizations to determine need and interest level. (HE)* - *Develop Complete Street policy (tracking), implementation…Action steps: Look for community interest for street audits. Hold focus groups…to determine needs, etc. (PA)* |
| **Build support and political will** | Encouraging advocates, policymakers, and the general public to support proposed policies.  [Adaptation made to the Lyn et al. framework definition] Strategy is assessed as aligned if a PSE solution has also been stated.   - *Work within municipalities and with key partners to identify ground to put in community gardens. (HE)* - *Prepare and engage the policy makers and public in addressing multi-modal methods of transportation in the next transportation plan as part of the growth policy. (PA)* |

Abbreviations: CHIP= community health improvement plan; HE= healthy eating; PA= physical activity
