# Supplementary material for: Healthy Eating and Physical Activity Policy, Systems, and Environmental Strategies: A Content Analysis of Community Health Improvement Plans
Source: Front Public Health. 2020 Dec 18;8:580175. doi: 10.3389/fpubh.2020.580175 (PMC7775553; doi:10.3389/fpubh.2020.580175)
Supplement: Supplementary file 2 [file Table_2.DOCX]

**Supplemental Digital Content Table 2. Fit statistics for latent class analyses among CHIPs containing PSE strategies by topic area**

| **# of classes** | **# of free parameters** | **Loglikelihood H0 value** | **AIC** | **BIC** | **Sample-size adjusted BIC** | **Entropy** |
| --- | --- | --- | --- | --- | --- | --- |
| ***PHYSICAL ACTIVITY*** | | | | | | |
| **Traditional latent class analysis model*** | | | | | | |
| 1 | 6 | -290.969 | 593.939 | 611.098 | 592.122 | - |
| 2 | 13 | -273.294 | 572.588 | 609.766 | 568.651 | 0.834 |
| 3 | 20 | -266.896 | 573.792 | 630.988 | 567.735 | 0.894 |
| 4 | 27 | -263.970 | 581.941 | 659.156 | 573.764 | 0.936 |
| 5 | 34 | -263.207 | 594.414 | 691.647 | 584.117 | 0.785 |
| **Nonparametric random effects multilevel latent class analysis models** | | | | | | |
| 1 | 13 | -273.295 | 572.589 | 609.767 | 568.652 | 0.837 |
| 2 | 15 | -263.448 | 556.896 | 599.793 | 552.353 | 0.953 |
| 3 | 17 | -263.447 | 560.895 | 609.512 | 555.746 | 0.952 |
| 4 | 19 | -263.448 | 564.896 | 619.232 | 559.141 | 0.679 |
| 5 | 21 | -263.448 | 568.896 | 628.952 | 562.536 | 0.784 |
| ***HEALTHY EATING*** | | | | | | |
| **Traditional latent class analysis model**^†^ | | | | | | |
| 1 | 6 | -161.460 | 334.919 | 348.904 | 329.991 | - |
| 2 | 13 | -152.177 | 330.354 | 360.645 | 319.677 | 0.847 |
| 3 | 20 | -148.561 | 337.122 | 383.736 | 320.695 | 0.828 |
| 4 | 27 | -145.786 | 345.572 | 408.502 | 323.395 | 0.896 |
| 5 | 34 | -143.744 | 355.489 | 434.734 | 327.563 | 0.783 |
| **Nonparametric random effects multilevel latent class analysis models** | | | | | | |
| 1 | 13 | -152.177 | 330.354 | 360.654 | 319.677 | 0.773 |
| 2 | 15 | -148.218 | 326.436 | 361.397 | 314.115 | 0.885 |
| 3 | 17 | -148.219 | 330.437 | 370.060 | 316.474 | 0.846 |
| 4 | 19 | -148.218 | 334.436 | 378.720 | 318.831 | 0.756 |
| 5 | 21 | -148.218 | 338.435 | 387.381 | 321.187 | 0.747 |

Abbreviations: AIC = Akaike information criterion; BIC = Bayesian information criterion

Notes: *Tests for physical activity 2 (H0) versus 3 classes: Vuong-Lo-Mendell-Rubing likelihood ratio test (p=0.0871), Lo-Mendell-Rubin adjusted LRT test (p=0.0919), and bootstrapped likelihood parametric likelihood ratio test (p = 0.0779); ^†^Tests for healthy eating 2 (H0) versus 3 classes: Vuong-Lo-Mendell-Rubing likelihood ratio test (p=0.3433), Lo-Mendell-Rubin adjusted LRT test (p=0.3517), and bootstrapped likelihood parametric likelihood ratio test (p = 1.0000).
